# HIV-1 Integrase Inhibition Activity by Spiroketals Derived from *Plagius flosculosus*, an Endemic Plant of Sardinia (Italy) and Corsica (France)

**DOI:** 10.3390/ph16081118

**Published:** 2023-08-08

**Authors:** Cinzia Sanna, Brigida D’Abrosca, Antonio Fiorentino, Federica Giammarino, Ilaria Vicenti, Angela Corona, Alessia Caredda, Enzo Tramontano, Francesca Esposito

**Affiliations:** 1 Department of Life and Environmental Sciences, University of Cagliari, Via Sant’Ignazio da Laconi 13, 09123 Cagliari, Italy; cinziasanna@unica.it; 2 Department of Environmental Biological and Pharmaceutical Sciences and Technologies, DiSTABiF University of Campania Luigi Vanvitelli, Via Vivaldi 43, 81100 Caserta, Italy; antonio.fiorentino@unicampania.it; 3 Department of Medical Biotechnologies, University of Siena, Viale Bracci 16, 53100 Siena, Italy; federica.giammari@gmail.com (F.G.); vicenti@unisi.it (I.V.); 4 Department of Life and Environmental Sciences, University of Cagliari, Cittadella Universitaria di Monserrato, SS554, 09042 Monserrato, Italy; angela.corona@unica.it (A.C.); alessia.caredda@unica.it (A.C.); tramon@unica.it (E.T.)

**Keywords:** *Plagius flosculosus*, ^1^H-NMR profiling, spiroketals, HIV-1 inhibitors, integrase, IN-LEDGF binding inhibitors

## Abstract

In this work we investigated, for the first time, the effect of *Plagius flosculosus* (L.) Alavi & Heywood, a Sardinian–Corsican endemic plant, on HIV-1 integrase (IN) activity. The phytochemical analysis of the leaves chloroform extract led us to isolate and characterize three compounds (SPK1, SPK2, and SPK3) belonging to the spiroketals, a group of naturally occurring metabolites of phytochemical relevance with interesting biological properties. Due to their structural diversity, these cyclic ketals have attracted the interest of chemists and biologists. SPK1, SPK2, and SPK3 were evaluated here for their ability to inhibit HIV-1 integrase activity in biochemical assays. The results showed that all the compounds inhibited HIV-1 IN activity. In particular, the most active one was SPK3, which interfered in a low molecular range (IC_50_ of 1.46 ± 0.16 µM) with HIV-1 IN activity in the presence/absence of the LEDGF cellular cofactor. To investigate the mechanism of action, the three spiroketals were also tested on HIV-1 RT-associated Ribonuclease H (RNase H) activity, proving to be active in inhibiting this function. Although SPK3 was unable to inhibit viral replication in cell culture, it promoted the IN multimerization. We hypothesize that SPK3 inhibited HIV-1 IN through an allosteric mechanism of action.

## 1. Introduction

The Human Immunodeficiency Virus (HIV-1), with its 38.4 million of infected peoples worldwide and 1.5 million new infections, still represents an important threat to public health [1].

HIV-1 is a retrovirus belonging to the Retroviridae family, with a viral single-strand RNA genome retrotranscribed into a double-stranded DNA by the HIV-1 Reverse Transcriptase (RT) enzyme. This enzyme possesses both RNA/DNA-dependent DNA polymerase and Ribonuclease H (RNase H)-associated functions [2]. After reverse transcription, the viral genome is integrated into the host DNA by HIV-1 integrase (IN), a 32 KDa encoded enzyme that, as the HIV-1 RT-associated RNase H, belongs to the polynucleotidyl transferase superfamily [3]. IN consists of three functional domains that allow the integration process through two successive distinct reactions named 3′-processing and strand-transfer [4]. The integration relies on the cooperation with a subset of specific viral and cellular proteins which form the viral preintegration complex (PIC) [5]. Among the cellular factors involved in the integration process, Lens epithelium-derived growth factor p75 (LEDGF/p75) is a cellular protein known to preferentially bind the tetrameric state of IN interacting with the binding site (IBD) located in the IN catalytic core domain (CCD) [5]. This interaction facilitates the integration of the viral genome retrotranscribed into the actively transcribed genes.

The latency and the establishment of viral reservoirs combined with the high variability of HIV-1 make it impossible to eradicate the HIV-1 infection and to develop an efficacious vaccine [6]. This highly stable reservoir of latently infected cells escapes the immune system and is not eradicated by currently available antiretroviral drugs [7]. 

However, the development and availability of highly effective combination antiretroviral therapy (cART), which consists of the combined administration of drugs belonging to five different classes targeting different steps of HIV-1 replication cycle, reduces HIV-1 mortality and improves the life quality of infected patients, contributing to transforming the HIV-1 infection from a fatal disease to a lifelong chronic and manageable infection. The success of cART is mainly due to its ability to suppress virus replication, reducing the viral load to levels undetectable in clinical assays. However, cART is not able to clear the infection, and the insurgence of resistance toward one or whole classes of drugs is an increasingly significant problem. For this reason, the identification of new antivirals with improved safety, tolerability and efficacy or innovative mechanisms of action is in high demand.

Among the different antivirals administered, the IN inhibitors (INIs) represent a relevant pharmacological class of drugs to treat HIV-1 infection. Since all approved INIs target the strand transfer (ST) reaction binding the IN active site, they are also called INSTIs. INIs have provided several advantages by offering minimal toxicity, daily dosing, and efficacy against viruses resistant to other drug classes. In addition, INIs are the only class that interacts with two components of the virus: IN and the viral DNA, which is the substratum for integration [8]. 

In the last 10 years, the research has been focused on the discovery of antiviral agents with innovative mechanisms of action, such as double HIV-1 IN and RT-associated RNase H inhibitors [9,10,11], or allosteric inhibitors [5,9,12,13,14]. This latter group includes different classes of compounds able to block the IN activity through different mechanisms, including interacting with IN-LEDGF/p75 in the binding site or IN–IN exchange, thereby inducing the oligomerization/multimerization of the enzyme and showing the different multimodal functionalities of HIV-1 IN [5,12,13,15,16,17,18,19].

In spite of several plant compounds exhibiting anti-HIV-1 activities, none have ever entered the list of antiretroviral drugs [20]. Owing to their structural and mechanistic diversity, plant-derived compounds could represent appealing scaffolds for the design and development of novel anti-HIV-1 IN drugs. 

In this context, in an effort to discover natural compounds able to block the HIV-1 infection, we focused our attention on the endemic flora of Sardinia Island (Italy), known to be an important source of bioactive molecules [21]. Compounds obtained from Sardinian endemic plants were found able to inhibit HIV-1 replication, blocking the HIV-1 IN LEDGF-dependent activity, or the IN–IN interaction and the RNase H function [22,23].

Pursuing our research on Sardinian endemic plants, in this study we focused on *Plagius flosculosus* (L.) Alavi & Heywood (Asteraceae), an endemic species of Sardinia and Corsica (France) [24].

To the best of our knowledge, only three reports have been published on the phytochemical characterization and biological properties of *P. flosculosus*. The essential oil from its flowers exhibited antimicrobial activity against *Candida albicans* (MIC = 250 µg/mL), and revealed (Z)-β-farnesene and β-phellandrene as the main components [25]. *P. flosculosus* was also described to be a rich source of diacetylenic spiroketal enol ethers, namely flosculin A-C, were identified for the first time from this species [26]. Polyacetylene compounds are characteristic of the Asteraceae family. Investigations of their bioactivity and biosynthesis have been reported in many articles. Some spiroketal enol ethers were found to be endowed with antibacterial activity against antibiotic-resistant *S. aureus* strains, due to their ability to inhibit biofilm formation and efflux pump activity [27]. Acetylenic spiroketal enol ethers also exhibited inhibitory effects against TPA-induced O_2_ generation in differentiated HL-60 cells [28] and proapoptotic properties in leukemia cells [26]. Some polyacetylene spiroketals from *P. flosculosus* were found to be able to inhibit NF-κB activation and the expression of inflammatory mediators [29]. Little is known in the literature regarding their antiviral activity: Alvarez et al. [30] reported their inhibition properties against *Herpes simplex* viruses (HSV-1 and HSV-2), but there are no previous studies on their inhibition of HIV-1 enzymes.

In this work we evaluated, for the first time, the potential anti-HIV-1 activity of three different extracts obtained from *P. flosculosus* leaves. Following a bio-guided approach, the most bioactive extract was further purified to obtain pure compounds, identified as diacetylenic spiroketal enol ethers. The antiviral activity of these compounds was first assessed in biochemical assays, evaluating the inhibition of HIV-1 IN activity in the presence and in the absence of the LEDGF cellular cofactor to hypothesize their mechanism of action. The compound possessing the highest activity in the biochemical assay was then evaluated in a cell-based assay.

## 2. Results and Discussion

### 2.1. Effect of P. flosculosus Extracts on HIV-1 IN Activity in the Presence of LEDGF/p75 Cellular Cofactor

**Figure 1:**
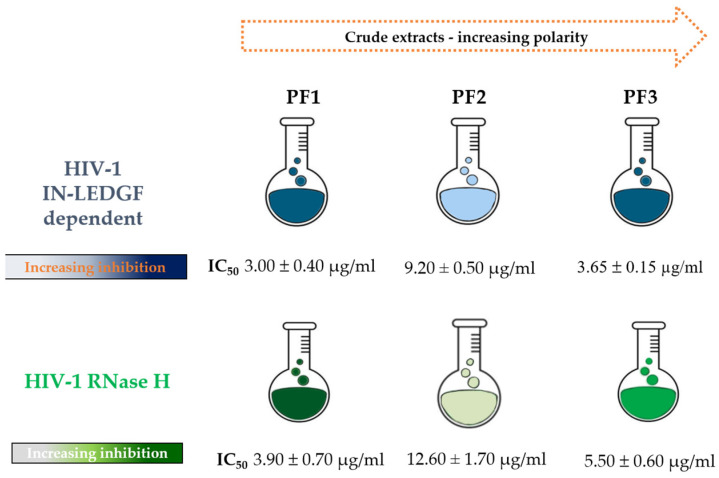
Inhibition of HIV-1 IN and RNase H activities of crude extracts of *P. flosculosus* tested in biochemical assays.

Aiming to find new bioactive plant compounds able to inhibit HIV-1, *P. flosculosus* leaves were sequentially extracted in solvents with increasing polarity (CHCl_3_, MeOH, MeOH/H_2_O 1:1) to obtain three crude extracts, namely, PF1, PF2, and PF3, respectively (Figure 1). All the crude extracts were screened on strand transfer LEDGF/p75-dependent IN activity. The results, shown in Figure 1, were expressed as IC_50_ values against IN LEDGF-dependent integration. Considering that an IC_50_ value up to 100 μg/mL is accepted as the threshold of activity for plant-derived mixtures in all anti-infective bioassays [31], all the extracts are worthy of note. The less polar extract (PF1) showed the highest inhibitory activity with an IC_50_ value of 3.00 ± 0.40 μg/mL. PF2 and PF3 also exhibited interesting inhibition effects, although less potent than PF1, with IC_50_ values ranging from 9.20 ± 0.50 to 3.65 ± 0.15 μg/mL, respectively. 

### 2.2. Effect of P. flosculosus Extracts on HIV-1 RT-Associated RNase H Activity

Given the structural homologies between HIV-1 IN and the RNase H catalytic core, a great number of plant extracts active on IN was found to be capable of affecting HIV-1 RNase H activity [22,23,32,33]. Therefore, with the aim to investigate whether *P. flosculosus* extracts exert dual inhibition on both HIV-1 enzymes, we evaluated their effect on HIV-1 RNase H function. The results, schematized in Figure 1, showed that all the extracts were able to reduce the HIV-1 RNase H activity, with IC_50_ values ranging from 3.90 to 12.60 µg/mL. According to the results, it emerged that all the extracts, particularly PF1, were able to interfere with both HIV-1 IN and RNase H activities but exhibited the highest inhibition on HIV-1 IN activity. 

### 2.3. Chemical Characterization of Bioactive Crude Extract

**Figure 2:**
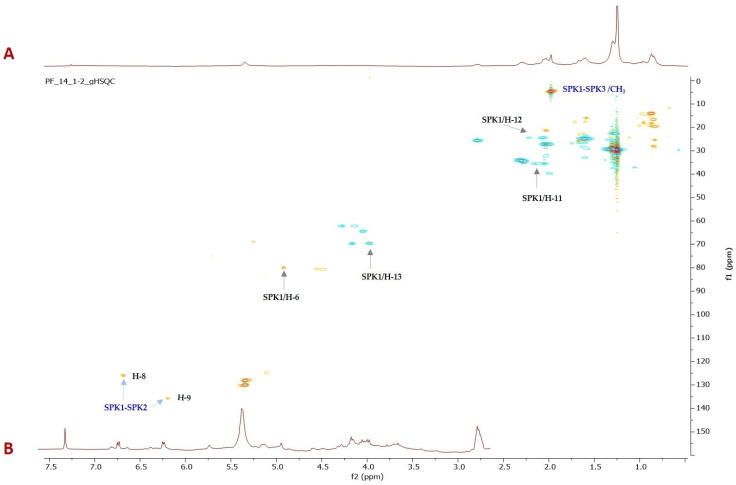
^1^H-^13^C HSQC (**A**) and expanded region of ^1^H-NMR (**B**) of chloroform crude extract (PF1). The multiplicity editing of the HSQC spectrum as shown (methine/methyl resonances have positive intensity and are plotted in red; methylene resonances, plotted in blue have negative intensity).

Although all the extracts exhibited potent inhibitory activity, we decided to phytochemically investigate the most active one (PF1). ^1^H-NMR profiling showed only two doublets in the olefinic region besides overlapped signals in the aliphatic region due to the presence of fatty acids (Figure 2). The above-mentioned doublets at δ_H_ 6.68 and 6.19 were, based on HSQC correlations (Figure 2), attributed to two methine carbons resonating at δ_C_ 126.0 and 135.8, respectively. Furthermore, in the same experiment, a methyl singlet at δ_H_ 1.97 linked to an upfield shifted carbon (δ_C_ 4.65) was also evident.

**Figure 3:**
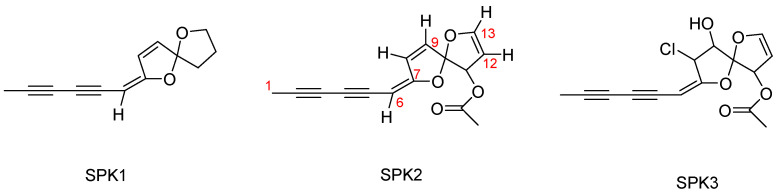
Chemical structures of SPK1–SPK3.

By implementing different chromatography methods, three diacetylenic spiroketal enol ethers (SPK1–SPK3) were first purified and then characterized using spectroscopic techniques (Figures S1–S5). Their chemical structures are shown in Figure 3. 

The NMR data are in accordance with the literature for tonghaosu (SPK1) and flosculin A (SPK3), already identified as constituents of *P. flosculosus* [26,29]. SPK1 and SPK2 have been also detected in *Matricaria recutita* L. [34] and *Santolina rosmarinifolia* L. [35], respectively. Table S1 reports NMR chemical shifts of the isolated SPK1–SPK3 compared with previously reported data.

Natural polyacetylenes are widely distributed in the Asteraceae family, and some of them are considered important chemotaxonomic markers since individual sets of acetylene metabolites characterize the chemical profile of various tribes of this family [36]. In particular, five-membered C_12_ spiroacetalenol esters are most widely represented in the Anthemideae tribe, which includes *Plagius* spp.

The polyacetylene spiroketals are characterized by two important centers of reactivity: a spiroketal moiety that contains at least two oxacyclic rings, and a 2,4-diine group (poliacetilenic component). As suggested by Calzado et al. [29], the exocyclic double bond of these compounds shows electrophilic properties and forms aromatic furan adducts by reacting with nucleophiles, resulting in opening of spiroketal moiety.

SPK1 displayed powerful antiphlogistic and insecticidal activities [37]. Some spiroketals isolated from *P. flosculosus* exhibited a strong anti-inflammatory activity by inhibiting the induction of NF-*k*B activity and the expression of different inflammatory mediators [29]. Moreover, a significant cytotoxicity of some spiroketals has been documented [26]. However, their anti-HIV-1 potential has not been reported yet.

### 2.4. Effects of Pure Compounds on HIV-1 IN Activity in the Presence of LEDGF/p75 Cellular Cofactor and on HIV-1 RNase H Activity

We evaluated the effects of SPK1–SPK3 on HIV-1 IN in the presence of LEDGF/p75 cofactor. As shown in Table 1, SPK1 and SPK3 potently inhibited the HIV-1 IN LEDGF-dependent activity, with IC_50_ values of 1.69 and 1.46 µM, respectively, while SPK2 produced a less marked inhibition (IC_50_ value of 14.70 µM). Interestingly, SPK1 and SPK3 showed a higher potency of HIV-1 IN inhibition compared with the crude extract PF1. 

As we previously reported [31], pure compounds often exhibited a weaker activity than crude extracts, and this might be due to a synergistic effect of the components in a phytocomplex [38,39,40]. 

Moreover, the spiroketals obtained from *P. flosculosus* showed an inhibitory effect on HIV-1 RNase H activity (Table 1). SPK1 and SPK3 inhibited the HIV-1 RNase H activity with IC_50_ values of 10.02 and 3.60 µM, respectively. SPK2, as observed for IN, exhibited a less potent inhibitory activity (IC_50_ value of 20.0 µM). Notably, our results showed a different pattern of inhibition of the two enzymes by the isolated compounds. RDS1759 and Raltegravir were used as positive controls for HIV-1 RNase H activity and HIV-1 IN-LEDGF-dependent activity, respectively. RDS1759 has been proven to inhibit RNase H involving interactions not only with Mg^2+^ but also with highly conserved residues within the RNase H domain [41]. Raltegravir is the first HIV-integrase inhibitor approved by the FDA for the treatment of HIV infection [42].

### 2.5. Pure Compounds’ Spectra in the Presence of Magnesium

Some compounds able to inhibit both HIV-1 IN and RNase H activities were reported to coordinate, in their active site, a divalent metal ion, which is an essential cofactor for their activity [9]. To verify this capability in our compounds, we performed a magnesium spectrum analysis, evaluating if SPK1–SPK3 were capable to chelate the divalent Mg^2+^ ion, similar to what was previously reported for the Diketo acid derivatives [43].

**Figure 4:**
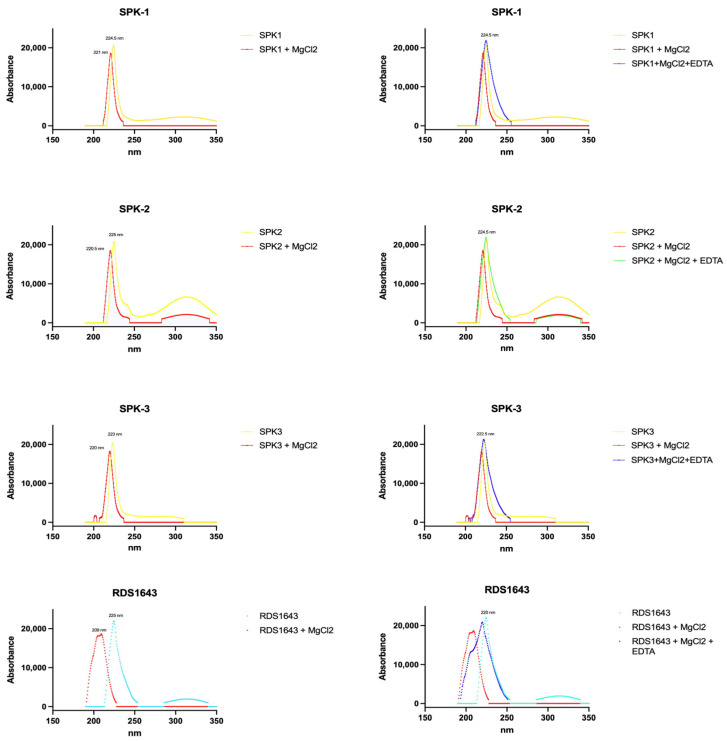
Effects of MgCl_2_ and MgCl_2_ with EDTA on the absorbance spectra of pure compounds (SPK1–SPK3) and RDS1643.

In contrast to the positive control Diketo acid RDS1643, which showed a significant shift of 16 nm in the maximum of absorbance in the presence of Mg^2+^ (Figure 4) because of chelation in the active site, all the studied compounds’ spectra did not show any expressive shift. In fact, the SPK1, SPK2 and SPK3 absorbance spectra only had shifts of 3.5 nm, 4.5 nm and 3 nm, respectively, suggesting a potential allosteric mechanism of action for these compounds.

### 2.6. Evaluation of Inhibition of Pure Compounds against HIV-1 IN Activities

Since SPK3 exhibited the highest inhibitory activity on both enzymes, it was selected for further experiments. To assess whether it inhibited HIV-1 IN through a mode of action different from that shown by the Diketo acid derivatives, we evaluated if its activity on HIV-1 IN was related to the interaction with LEDGF/p75 protein by performing IN activity measurements in the absence of this protein. As shown in Table 2, SPK-3 was also able to inhibit HIV-1 IN in the absence of the LEDGF/p75 cellular cofactor but with an 18.7-fold decrease in potency.

It is known that after the binding with the DNA, HIV-1 IN exerts a conformational change and LEGDF/p75 can modulate the IN–DNA complex [44,45]. In this study, LEDGF/p75 interacts with IN through its specific domain, and we hypothesize that its binding to IN may change the complex conformation, affecting the binding ability of SPK3.

In addition, since IN acts as a multimer, we evaluated the ability of SPK3 to block the different protein–protein interactions by testing whether it affected the IN multimerization process. As shown in Table 2, SPK3 induced higher-order aberrant IN multimerization, with an IC_50_ value of 1 µM, without any effect on the IN–IN subunit exchange, similarly to Kuwanon-L, which was used as a control (Table 2). Differently from the Kuwanon-L, which inhibits HIV-1 IN activities by acting on the sucrose binding site with an allosteric mechanism of action, unexpectedly, SPK3 did not inhibit the binding with HIV-1 IN and LEDGF cellular cofactor, showing a different behavior with respect to the control.

### 2.7. Inhibition of HIV-1 Replication in a Cell-Based Assay by SPK3

**Figure 5:**
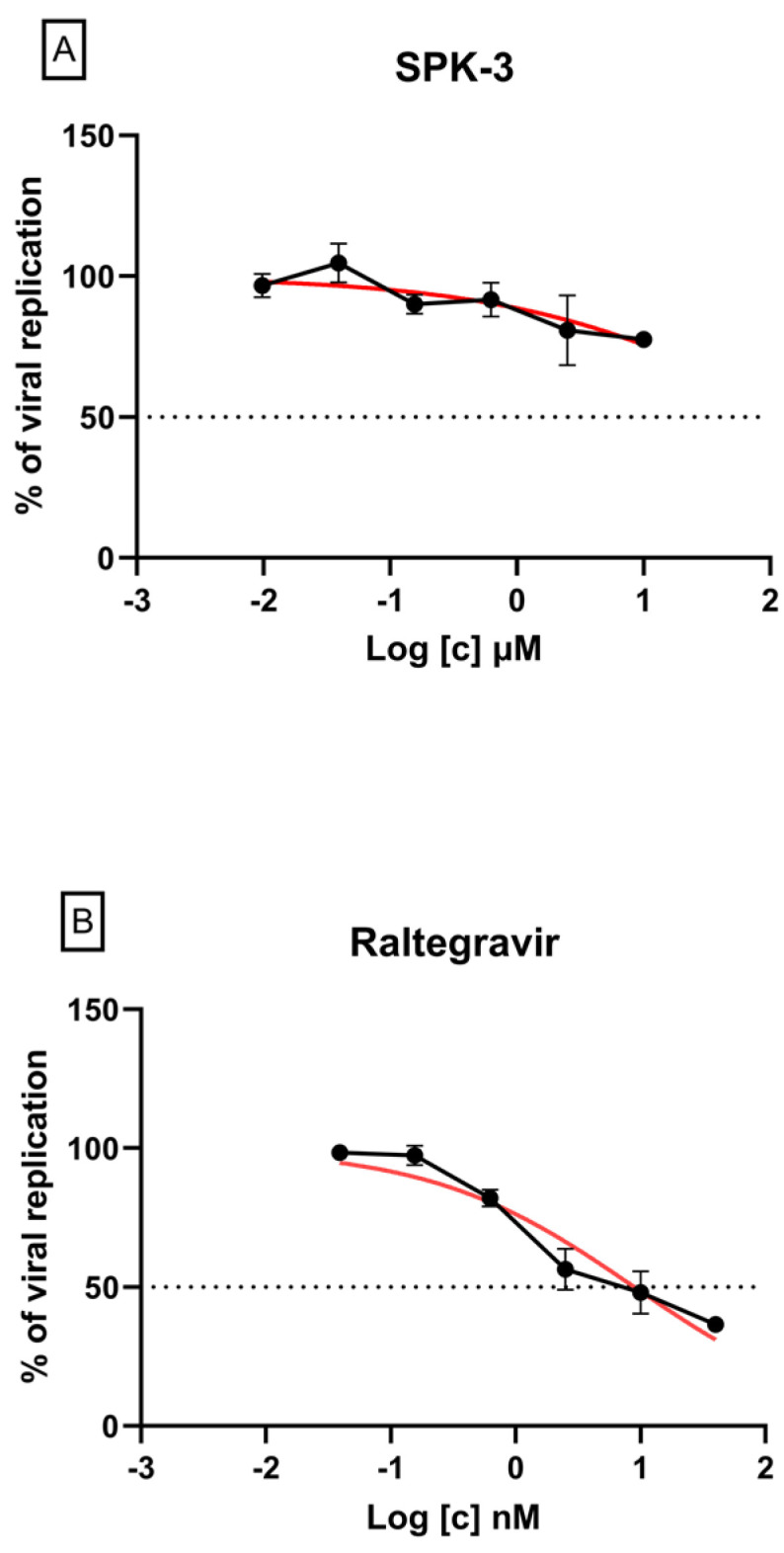
Normalized antiviral activity of SPK3 (**A**) and Raltegravir (**B**) as determined against NL4-3 HIV-1 wild-type strain in TZM-bl cell line. The nonlinear fitting curve as calculated by GraphPad Prism is depicted in red; the black lines indicate the dose-response curves. The R^2^ of nonlinear fitting curve is 0.913 for Raltegravir and 0.631 for SPK-3.

The SPK3 efficacy against HIV-1 was tested in vitro in a cell-based assay evaluating the inhibition of viral replication. The cytotoxicity of SPK3 was evaluated in the reporter cell line TZM-bl quantifying the cell viability; in the antiviral assays, the non-toxic dose corresponding to 90–100% cell viability was used as the maximum concentration. As shown in Table 3, SPK3 did not inhibit the HIV-1 replication (Figure 5A) and displayed toxic effects with a half-maximal cytotoxic concentration (CC_50_) of 50 µM (Table 3). The values of EC_50_ obtained for the reference compound Raltegravir (Figure 5B) were consistent with those previously published [46]. 

## 3. Materials and Methods

### 3.1. Plant Material and Sample Preparation

Leaves of *P. flosculosus* were sampled during the blooming period (July 2020) at the site of Marina di Cardedu (Sardinia, Italy, 39°46′56.3″ N 9°39′37.1″ E). The plant was authenticated by Cinzia Sanna, and a sample was deposited at the General Herbarium (*Herbarium*CAG) of the University of Cagliari with the voucher specimen CAG 743/V1. Even though endemic, the plant is not under the protection of national or international regulations, so that it was not necessary to obtain specific permission for its harvesting. The leaves were dehydrated at 40 °C using a ventilated stove, and powdered with an electric grinder.

### 3.2. Preparation of Crude Extracts for Bioassay

The powdered leaves (30 g) were sequentially and exhaustively extracted by ultrasound assisted extraction (Branson 3800 MH) for 40 min each (2 cycles × 3.0 L) with chloroform, MeOH, and MeOH:H_2_O (1:1). After centrifugation at 4800 rpm (Beckman, GS-15R) at 4 °C for 10 min, the solutions were concentrated under vacuum, giving three crude extracts: chloroform (PF1, 2.2 g), MeOH (PF2, 4 g), and MeOH:H_2_O (PF3, 1.6 g).

### 3.3. Bioguided-Fractionation of the Active Extract and Compounds Purification

#### 3.3.1. General Chromatographic Procedures

Analytical and preparative TLC were performed as described by Guzzo et al. [22]. Column chromatography (CC) was performed on Sephadex LH-20, on Merck (Milan, Italy) Kieselgel 60 (70–240 µm) and Merck Kieselgel 60 (40–63 µm).

#### 3.3.2. NMR Experiments

NMR spectra were recorded at 25 °C at 300.03 MHz for ^1^H and at 75.45 MHz for ^13^C on a Bruker AVANCE II 300 MHz NMR Spectrometer Fourier transform in CD_3_OD or CDCl_3_ (Bruker, Billerica, MA, USA). Chemical shifts are reported in δ (ppm) and referenced to the residual solvent signal; *J* (coupling constant) is given in Hz. 

^1^H-NMR and ^13^C NMR spectra were acquired using the standard Bruker parameter already described by Guzzo et al., 2023 [47]. Set pulse sequences for homonuclear and heteronuclear 2D-NMR experiments are present in Bruker’s Library, as already reported by Guzzo et al. [47]. 

#### 3.3.3. Compound Purification

The chloroform crude extract (PF_1_) was chromatographed by CC Sephadex LH-20 column and eluted with hexane/CHCl_3_/CH_3_OH (3:1:1). The three fractions obtained were then later purified by preparative TLC (1 mm) with hexane:EtOAc (8:1) as eluent, giving SPK1 (10.0 mg), SPK2 (7.0 mg) and SPK3 (4.0 mg) in pure form.

The compounds were stored lyophilized and resuspended in DMSO before testing in the assays. 

### 3.4. Anti-HIV Biochemical Assays

#### 3.4.1. Recombinant Proteins Preparation

Recombinant 6xHis IN protein, FLAG-IN, His-LEDGF and FLAG-LEDGF were expressed in *Escherichia coli* strain BL21 (DE3) and purified as previously described [9]. His-tagged p66/p51 HIV-1 RT was expressed in *E. coli* strain M15 and purified as previously described [43].

#### 3.4.2. HTRF LEDGF-Dependent and -Independent Assays

The evalaution of 3′-processing and strand-transfer IN reactions in the presence or in the absence of recombinant LEDGF/p75 cellular cofactor were performed as described previously [12]. Briefly, recombinant IN was pre-incubated for 1 h at room temperature, in a buffer containing 20 mM HEPES pH 7.5, 1 mM DTT, 1% Glycerol, 20 mM MgCl_2_, 0.05% Brij-35 and 0.1 mg/mL BSA and increasing concentrations of the tested compounds.

Then, 9 nM Biotin3′-DNA donor substrate 50 nM 5′-Cy5-DNA acceptor substrate and 50 nM LEDGF/p75 protein (when required) were added to this reaction and incubated at 37 °C for 90 min. After the addition of 4 nM of Europium-Streptavidin to the reaction mixture and a second incubation, the HTRF signal was recorded at 314 nm for the excitation wavelength and at 668 and 620 nm for the wavelength of the acceptor and the donor substrate emissions, respectively, using a plate reader (Perkin Elmer Victor 3).

All the oligos used for the experiments were custom products and were synthetised and purified by FPLC from Metabion (Planegg, Germany).

#### 3.4.3. IN–LEDGF Binding Assay

Recombinant His-IN was pre-incubated for 30 min at room temperature in the presence of the tested compounds in a mixture reaction composed of 150 mM NaCl, 2 mM MgCl_2_, 0.1% Nonidet P-40, 1 mg/mL BSA, and 25 mM Tris pH 7.4 [12,48]. After this time, FLAG-LEDGF was added and the mixture was incubated for 30 min at room temperature. A mixture of two specific antibodies (anti-His6-XL665 and anti-FLAG-EuCryptate) was then added to the reaction and incubated for 4 h at 4 °C. The HTRF signal was recorded as previously described, and the HTRF signal was expressed as the emission ratio 665 nm/620 nm multiplied by 10,000.

#### 3.4.4. IN–IN Binding Assay

This IN–IN binding assay allows the verification of higher-order, aberrant IN multimerization or IN–IN subunit exchange. Briefly, His and FLAG-tagged IN enzymes were added in a buffer containing 25 mM Tris pH 7.4, 150 mM NaCl, 2 mM MgCl_2_, 0.1% Nonidet P-40, and 1 mg/mL BSA [12,48] with different concentrations of compounds, and incubated for 2.5 h at room temperature. After this time, a mixture of two specific antibodies (anti-His6-XL665 and anti-FLAG-EuCryptate) was added to the reaction and incubated at room temperature for 3 h. The HTRF signal was recorded as described in the previous paragraph.

The analysis of the signal in order to evaluate the aberrant IN multimerization and interference with the IN–IN subunit exchange or functional IN–IN interactions was performed as described above.

#### 3.4.5. RT-Associated RNase H Assay

The HIV-1 RNase H activity was determined as described by Esposito et al. [9]. Briefly, 100 µL of volume of reaction containing 50 mM Tris HCl pH 7.8, 6 mM MgCl_2_, 1 mM DTT, 80 mM KCl, hybrid RNA/DNA (5′-GTTTTCTTTTCCCCCCTGAC-3′-Fluorescein, 5′-CAAAAGAAAAGGGGGGACUG-3′-Dabcyl), and 3.8 nM RT was incubated for 1 h at 37 °C. The reaction was stopped by the addition of EDTA and measured with a plate reader at 490/528 nm [48].

#### 3.4.6. Compound Spectra in the Presence of MgCl_2_

The ability of compounds to chelate the MgCl_2_ was measured using 15% ethanol and 15 mM Tris HCl pH 7.8 (in 1 mL of volume). The UV–VIS spectrum was recorded, before and after the addition of 6 mM MgCl_2_, from 250 nm to 600 nm.

### 3.5. Cytotoxicity Assay

Cytotoxicity was determined by the CellTiter-Glo 2.0 Luminescent Cell Viability Assay (Promega) according to the manufacturer’s protocol. Briefly, 15,000 cells/well of Tzm-bl were exposed to serial diluition of the drug and after 48 h, cell viability was calculated by measuring cellular ATP through a luciferase-based chemical reaction. The luminescent signal obtained from cells treated with serial dilution of the tested compounds, or the control (DMSO), was measured using the GloMax^®^ Discover Multimode Microplate Reader (Promega) and processed with GraphPad PRISM software to calculate the half-maximal cytotoxic concentration (CC_50_). The DMSO curve, used as a control, was added in each plate and SPK-3 was tested in duplicate. The non-toxic dose corresponding to 90–100% cell viability was used as the maximum concentration in the antiviral assays.

### 3.6. Determination of Anti-HIV-1 Activity in TZM-bl Cell Line

The antiviral activity of the investigated compound was evaluated by measuring the IC_50_ values against the HIV-1 wild-type reference strain NL4-3 in a TZM-bl cell line-based phenotypic assay, as previously published [46]. Briefly, 15,000 cells/well were infected with the wild-type NL4-3 strain at multiplicity of infection (MOI) of 0.1 in the presence of serial diluitions of the drug in a 96-well plate format. In each plate, the raltegravir used as a reference compound (MedChem Express, code HY-10353), the mock control (uninfected cells) and the virus control were included. Each compound was tested in duplicate and tested in two independent experiments. After 48 h, the cells were detached with the Glo-Lysis buffer (Promega, Madison, WI, USA), and the Bright-Glo Luciferase reagent (Promega) was added to each well as indicated by the manufacturer. Finally, the relative luminescence units were measured using the GloMax Discover instrument (Promega) as previously described and processed with GraphPad software to calculate half-maximal inhibitory concentration (IC_50_) values.

## 4. Conclusions

This research into new plant-derived compounds allowed us to successfully identify three spiroketals derived from the Sardinian–Corsican endemic species *P. flosculosus*, which are capable of blocking HIV-1 IN activities and, with less potency, also the HIV-1 RT-associated RNase H function. More precisely, SPK3 potently inhibited HIV-1 IN activity in the presence/absence of the LEDGF/p75 cellular cofactor and promoted the IN multimerization in a low molecular range, showing a different profile compared with the known active site and allosteric inhibitors. SPK3 showed no activity in a cell-based assay using living virus, which is considered as the gold standard for antiviral screening. To exclude possible activity in the biological cell-based assay, further experiments using different cell lines and different timepoints will be conducted. Moreover, future experiments will be performed with multiple cycles of infection to exclude the effects of compounds’ activity in a late step of HIV-1 infection. Therefore, SPK3 could represent an attractive new scaffold for the development of new chemical drugs that might be active in the nanomolar range on HIV-1 IN and RNase H activities and, by reducing its cytotoxicity, also on HIV-1 viral replication.

## Figures and Tables

**Table 1:** Inhibition of HIV-1 IN and RNase H activities of pure compounds SPK1–SPK3 isolated from *P. flosculosus* tested in biochemical assay.

Compound	IN-LEDGF-Dependent ^1^ IC_50_ (µM)	RNase H ^1^ IC_50_ (µM)
SPK1	1.69 ± 0.38	10.02 ± 5.40
SPK2	14.70 ± 0.30	20.0 ± 6.10
SPK3	1.46 ± 0.16	3.60 ± 0.60
Raltegravir	0.05 ± 0.02	-
RDS1759	-	0.0067 ± 0.90

^1^ Compound concentration required to inhibit the catalytic activities of HIV-1 IN and RNase H function by 50%. IC_50_ was expressed as means ± standard deviation of three independent experiments.

**Table 2:** Inhibitory activity of SPK3 derived from *P. flosculosus* on HIV-1 integrase–LEDGF binding, integrase–integrase, and integrase LEDGF-independent activity.

Code	IN–LEDGF Binding ^1^ IC_50_(µM)	IN–IN Subunit Exchange ^2^ IC_50_(µM)	IN Multimerization ^3^ MI_50_(µM)	LEDGF-Independent IN Activity ^4^ IC_50_(µM)
SPK3	>100	>100	1.00 ± 0.01	27.3 ± 2.3
Kuwanon-L	22.0 ± 0.5	>100	38.0 ± 0.02	34.0 ± 0.5

^1^ Compound concentration required to inhibit the HIV-1 IN LEDGF interaction by 50%. ^2^ Compound concentration required to inhibit the HIV-1 IN–IN subunit exchange by 50%. ^3^ Compound concentration required to inhibit the multimerization increase by 50%. ^4^ Compound concentration required to inhibit the HIV-1 IN catalytic activities by 50% in the absence of LEDGF. IC_50_ was expressed as means ± standard deviation of three independent experiments.

**Table 3:** SPK-3 activity and cytotoxicity against HIV-1 in TZM-bl cell line.

Compound	^1^ EC_50_ (µM)	^2^ CC_50_ (µM)
SPK3	NA	50.1 ± 4.3
Raltegravir	0.0087 ± 0.059	>100

^1^ EC_50_: half-maximal compound concentration inhibiting HIV-1 viral replication. EC_50_ was expressed as means ± standard deviation of two independent experiments. ^2^ CC_50_: half-maximal compound cytotoxic concentration.

## Data Availability

The data presented in this study are available within this article.

## Supplementary Materials

The following supporting information can be downloaded at: https://www.mdpi.com/article/10.3390/ph16081118/s1. Figure S1: ^1^H-NMR of compound SPK1; Figure S2: ^1^H-^13^C HSCQ of compound SPK1; Figure S3: ^1^H-NMR of compound SPK2; Figure S4: ^13^C-NMR of compound SPK2; Figure S5: ^1^H-NMR of compound SPK3; Table S1: NMR data of compounds SPK1–SPK3 recorded in CDCl_3_ compared with NMR data reported for SPK1 [34], SPK2 [35], and SPK3 [26].
