# Supplementary material for: HIV-1 Integrase Inhibition Activity by Spiroketals Derived from Plagius flosculosus, an Endemic Plant of Sardinia (Italy) and Corsica (France)
Source: Pharmaceuticals (Basel). 2023 Aug 8;16(8):1118. doi: 10.3390/ph16081118 (PMC10457970; doi:10.3390/ph16081118)
Supplement: Supplementary file 1 [file pharmaceuticals-16-01118-s001.zip › pharmaceuticals-2531045-supplementary.pdf]

Figure S1: ^{1}H-NMR of compound SPK1

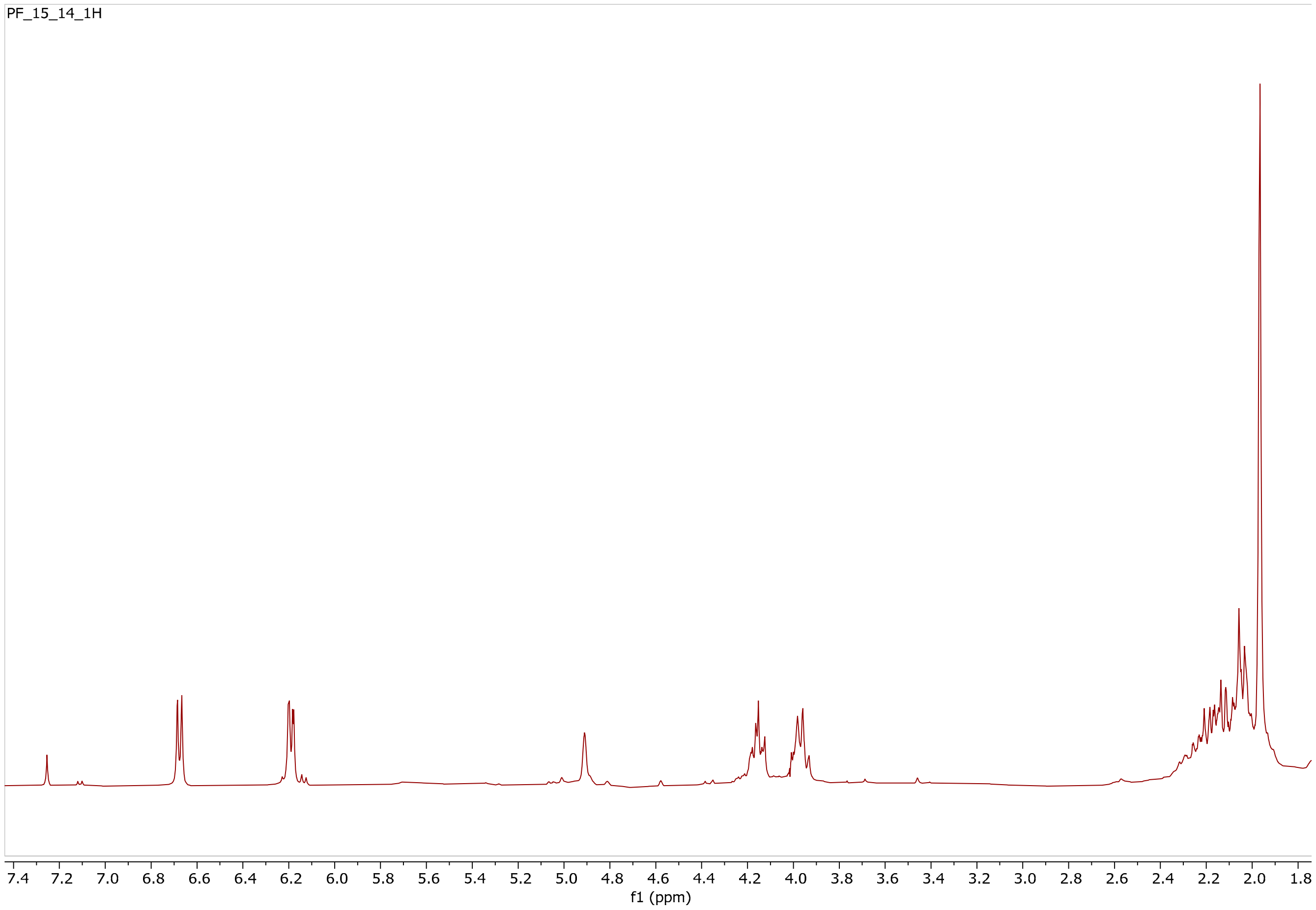

Figure S2: ^{1}H-^{13}C HSCQ of compound SPK1

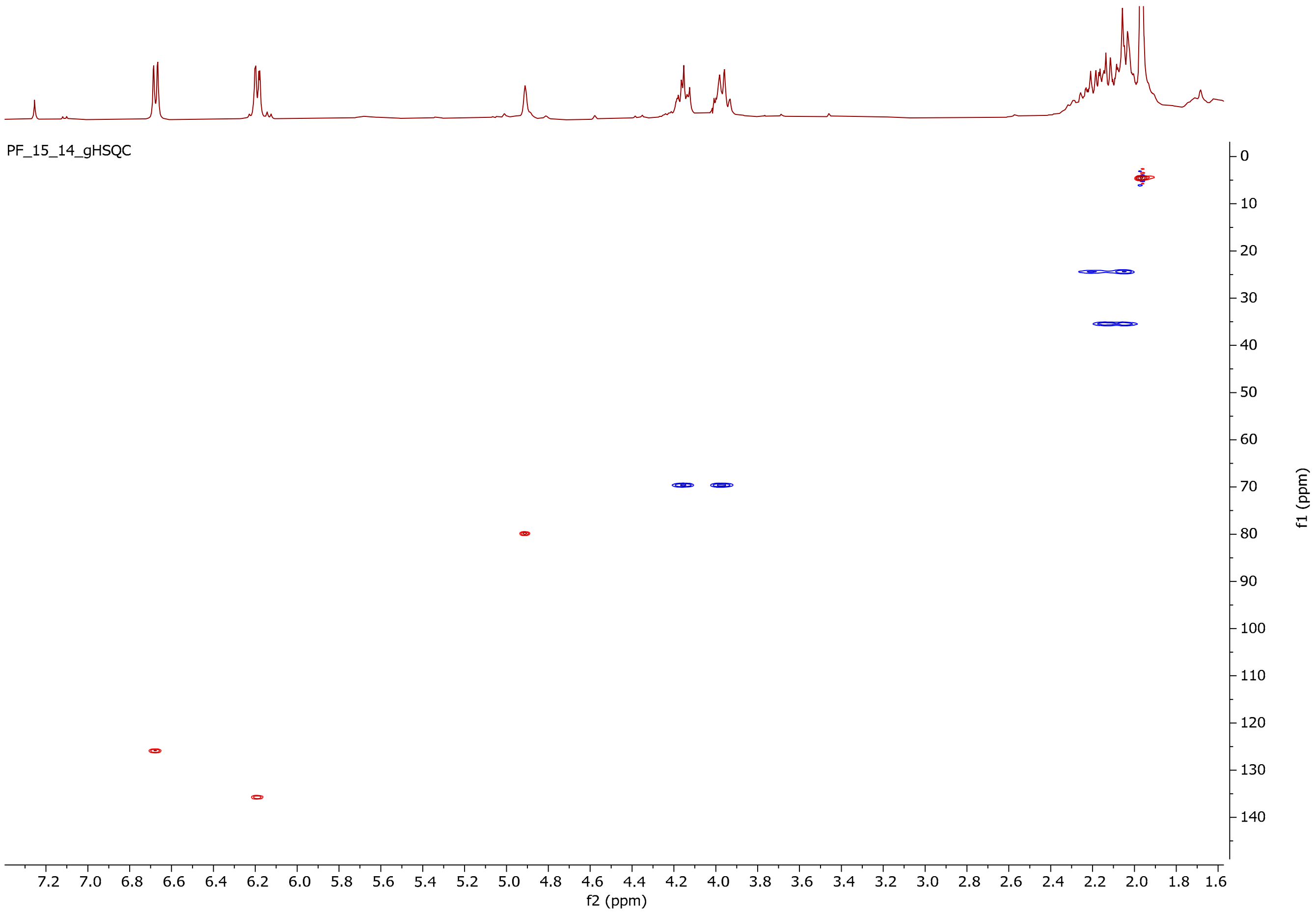

Figure S3: ^{1}H-NMR of compound SPK2

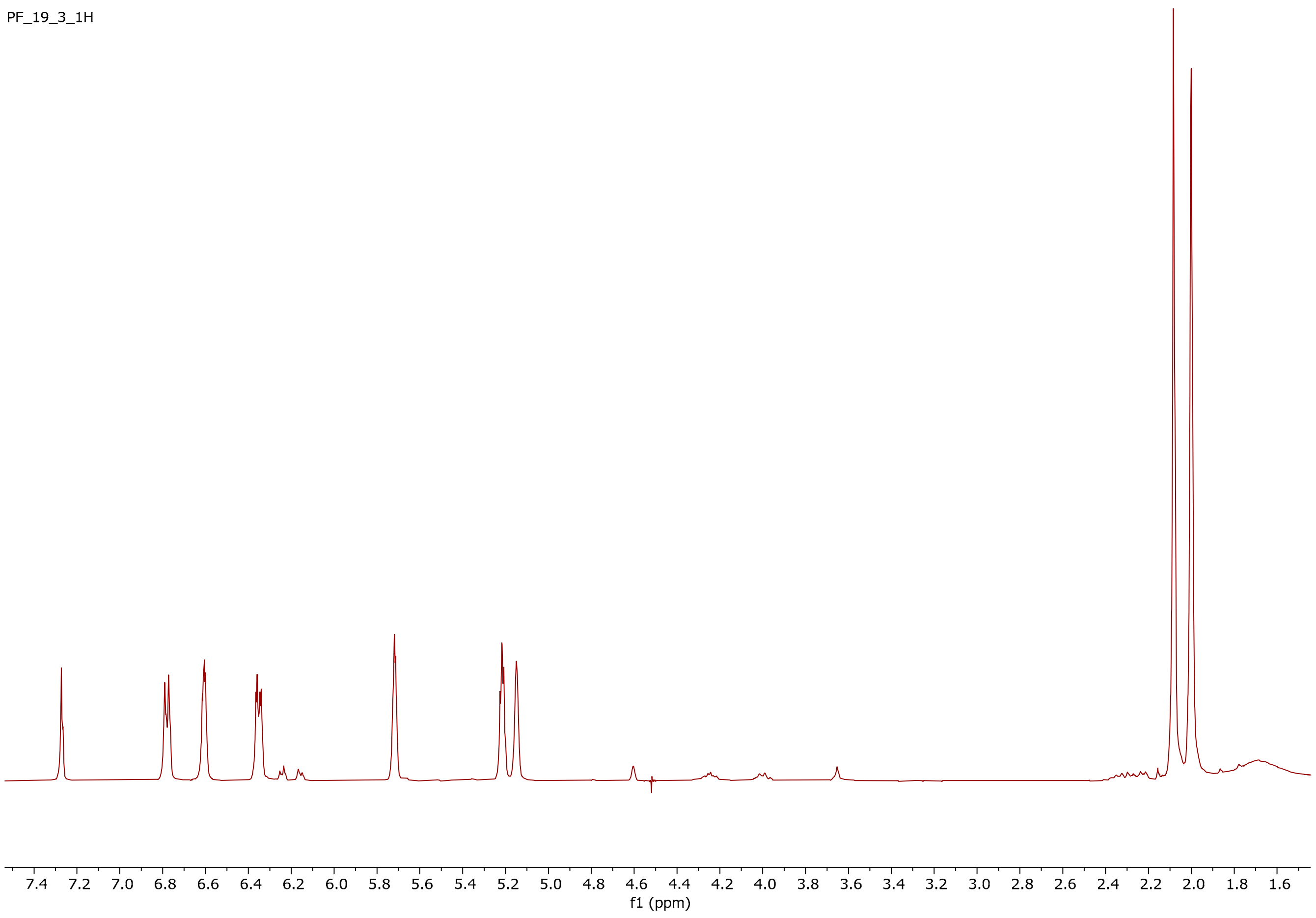

Figure S4: ^{13}C-NMR of compound SPK2

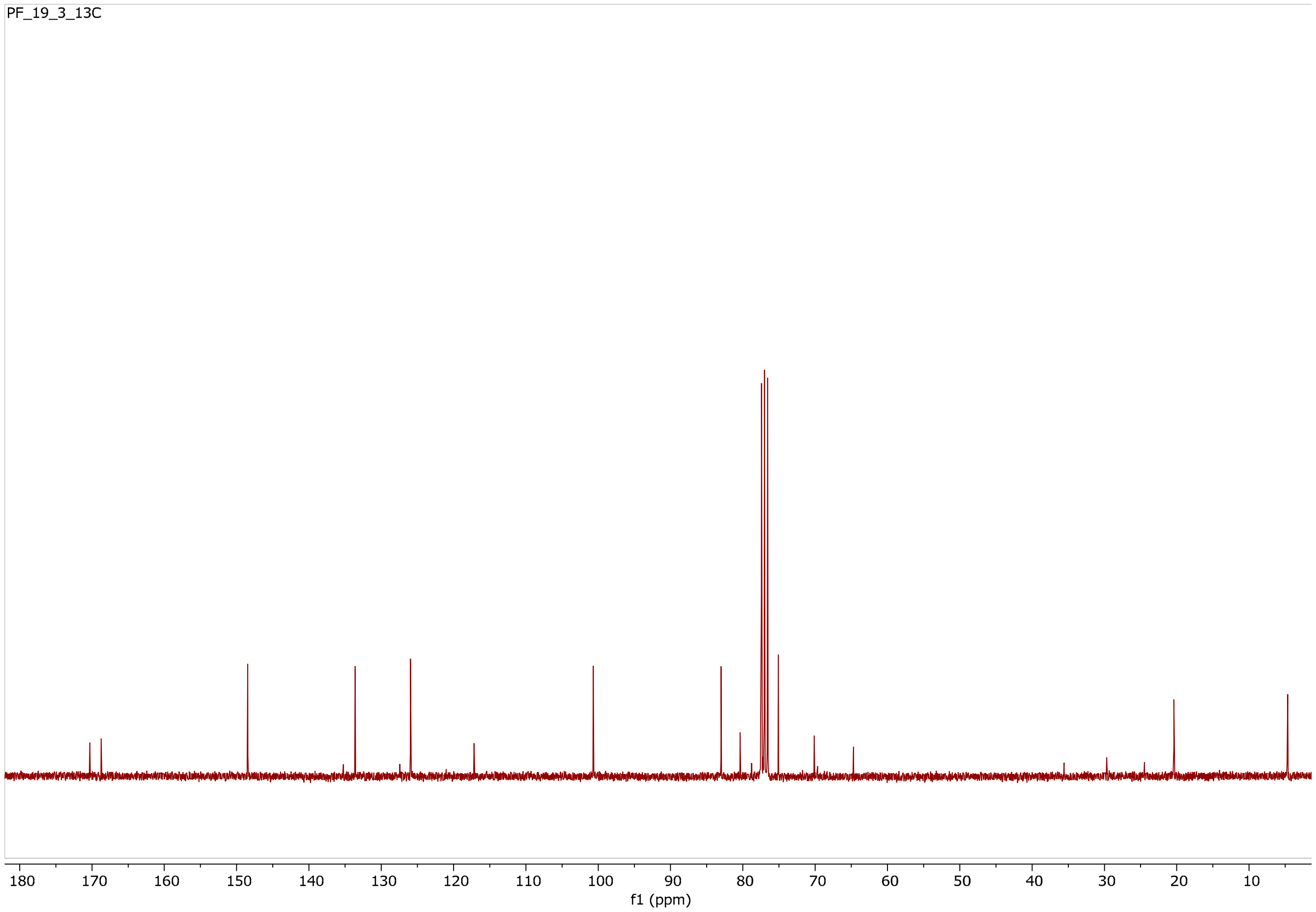

Figure S5: ^{1}H-NMR of compound SPK3

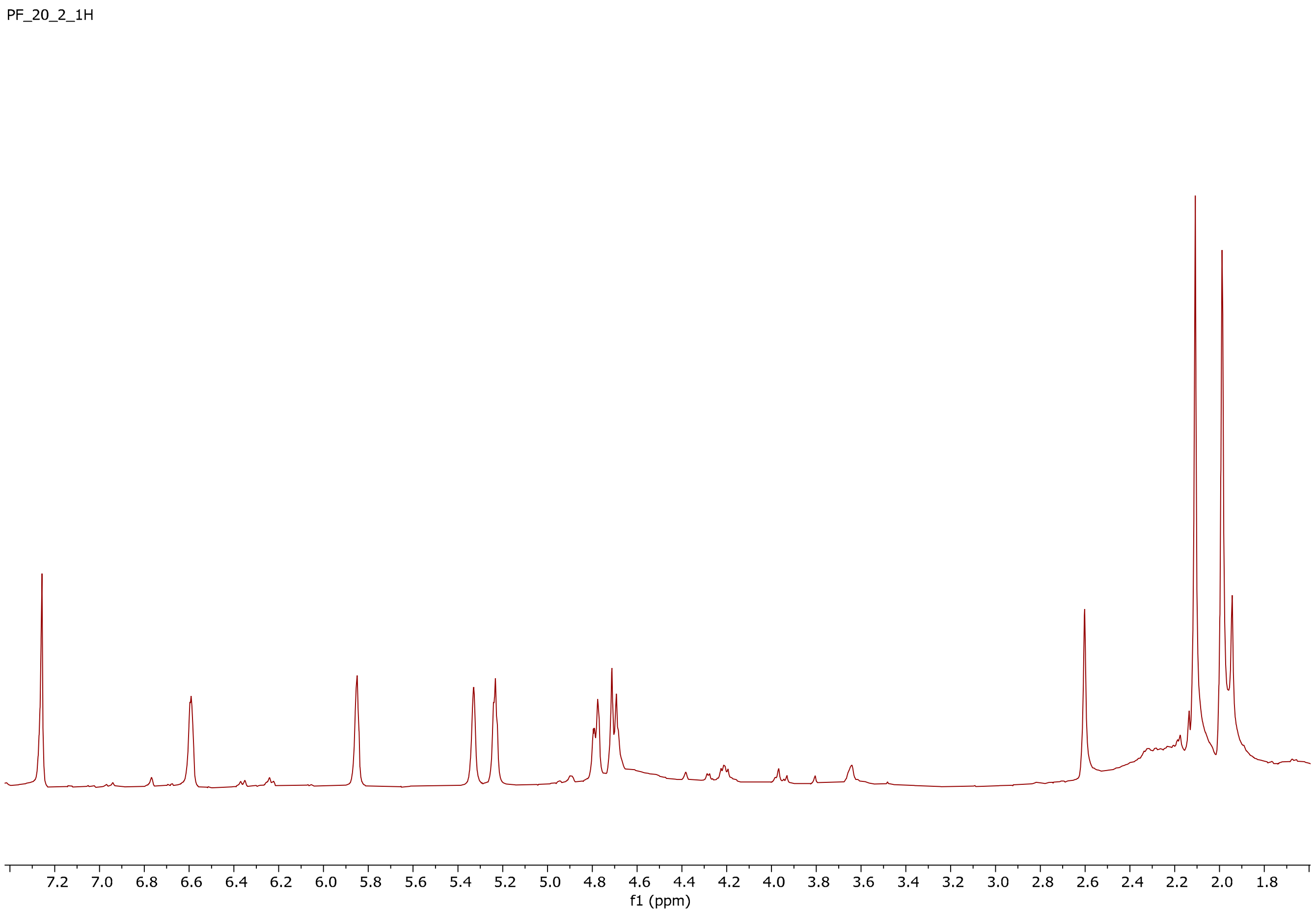

Table S1. NMR data of compounds SPK1-SPK3 recorded in $CDCl_3$ compared with NMR data reported for SPK1 [34], SPK2 [35], SPK3 [26].

	SPK1				SPK2				SPK3			
**Position**	**δ_C (type)**	**δ_H (J in Hz)**	**δ_C (type)[1] [34]**	**δ_H[1] (J in Hz)**	**δ_C (type)**	**δ_H (J in Hz)**	**δ_C (type)[2] [35]**	**δ_H[2] (J in Hz)**	**δ_C (type)**	**δ_H (J in Hz)**	**δ_C (type)[3] [26]**	**δ_H[3] (J in Hz)**
**1**	4.53 (CH_3)	1.97 s	5.0	1.98 s	4.78 (CH_3)	1.99 s	4.03	1.98 s	4.53 (CH_3)	1.97 s	4.0	1.48 s
**2**	79.9 (C)	-	80.0	-	80.4 (C)	-	80.15	-	79.9 (C)	-	82.5	-
**3**	65.0 (C)	-	65.4	-	64.7 (C)	-	64.52	-	64.8 (C)	-	65.7	-
**4**	76.3 (C)	-	76.9	-	77.5(C)	-	77.27	-	76.3 (C)	-	81.6	-
**5**	71.3 (C)	-	71.9	-	70.1 (C)	-	69.88	-	67.5 (C)	-	68.3	-
**6**	79.9 (CH)	4.91 brs	80.3	4.92 s	83.0 (CH)	5.14 brs	82.15	5.13 brs	87.0 (CH)	5.30 br s	87.7	5.39 brs
**7**	168.9 (C)	-	169.2		168.7 (C)	-	168.54		164.7 (C)	-	164.9	-
**8**	125.9 (CH)	6.68 d (5.7)	126.3	6.68 d (5.9)	125.9 (CH)	6.77 d (5.4)	125.27	6.76 d (5.7)	57.3 (CH)	4.78 d (6.0)	57.7	4.69 dd (1.8, 6.5)
**9**	135.8 (CH)	6.20 dd (1.8, 5.7)	136.2	6.21 dd (1.71, 5.9)	133.6 (CH)	6.34 dd (1.8, 5.4)	133.79	6.34 dd (1.8, 5.7)	80.8 (CH)	4.68 d (6.0)	81.4	4.73 brs
**10**	120.6 (C)	-	121.2	-	117.2 (C)	-	116.86	-	110.5 (C)	-	110.7	-
**11**	35.5(CH_2)	2.04 ov 2.15 ov	35.9	2.07-2.13 m	75.1 (CH)	5.70 brs	74.74	5.70 dd (2.0, 2.0)	75.4 (CH)	5.82 brs	35.9	2.07-2.13 m
**12**	24.5(CH_2)	2.05 ov 2.20 ov	24.9	2.07-2.17 m	100.7 (CH)	5.20 dd (2.7, 3.0)	100.38	5.20 dd (2.8, 2.8)	101.9 (CH)	5.20 brs	24.9	2.07-2.17 m
**13**	69.8(CH_2)	3.97 m 4.16 m	70.1	3.98-4.17 m	148.5 (CH)	6.59 dd (1.8, 3.0)	148.03	6.59 dd (3.1, 3.1)	147.9 (CH)	6.58 brs	70.1	3.98-4.17 m
**OCO<u>CH</u>$_3$**	-	-	-	-	20.4 (CH_3)	2.07 s	19.79(CH_3)	2.06 s	20.5(CH_3)	2.10 s	19.9(CH_3)	1.72 s
**O<u>CO</u>CH$_3$**	-	-	-	-	170.3 (C)	-	169.66 (C)		170.7 (C)	-	170.2 (C)	-

brs = broad singlet; d = doublet; dd = doublet of doublets; m = multiplet; ov = overlapped; s = singlet; δ in ppm; *J* values (Hz) are reported in brackets

[1] as reported by Buono-Core et al. 2011 [34]

[2] as reported by Maqua et al. 1988 [35]

[3] as reported by Casu et al. 2006 [26]
